# Comparative Transcriptome Analysis on the Regulatory Mechanism of Thoracic Ganglia in *Eriocheir sinensis* at Post-Molt and Inter-Molt Stages

**DOI:** 10.3390/life12081181

**Published:** 2022-08-03

**Authors:** Meiyao Wang, Jun Zhou, Shengyan Su, Yongkai Tang, Gangchun Xu, Jianlin Li, Fan Yu, Hongxia Li, Changyou Song, Meng Liang, Jingjing Jiang, Pao Xu

**Affiliations:** 1 Key Laboratory of Freshwater Fisheries and Germplasm Resources Utilization, Ministry of Agriculture and Rural Affairs, Freshwater Fisheries Research Center, Chinese Academy of Fishery Sciences, Wuxi 214081, China; wangmy@ffrc.cn (M.W.); susy@ffrc.cn (S.S.); lijl@ffrc.cn (J.L.); yuf@ffrc.cn (F.Y.); lihx@ffrc.cn (H.L.); songcy@ffrc.cn (C.S.); xup@ffrc.cn (P.X.); 2 Wuxi Fisheries College, Nanjing Agricultural University, Wuxi 214081, China; 2020113016@stu.njau.edu.cn (M.L.); 2020113015@stu.njau.edu.cn (J.J.); 3 Freshwater Fisheries Research Institute of Jiangsu Province, Nanjing 210017, China; finedrizzle@163.com

**Keywords:** *Eriocheir sinensis*, post-molt, inter-molt, thoracic ganglia, signal transduction

## Abstract

*Eriocheir sinensis* is an aquatic species found distributed worldwide. It is found in the Yangtze River of China, where the commercial fishing of this valuable catadromous aquatic species has been banned. As an important member of the phylum Arthropoda, *E. sinensis* grows by molting over its whole lifespan. The central nervous system of *Eriocheir sinensis* plays an important regulatory role in molting growth. Nevertheless, there are no reports on the regulatory mechanisms of the nervous system in *E. sinensis* during the molting cycle. In this study, a comparative transcriptome analysis of *E. sinensis* thoracic ganglia at post-molt and inter-molt stages was carried out for the first time to reveal the key regulatory pathways and functional genes operating at the post-molt stage. The results indicate that pathways and regulatory genes related to carapace development, tissue regeneration, glycolysis and lipolysis and immune and anti-stress responses were significantly differentially expressed at the post-molt stage. The results of this study lay a theoretical foundation for research on the regulatory network of the *E. sinensis* nervous system during the post-molt developmental period. Detailed knowledge of the regulatory network involved in *E. sinensis* molting can be used as a basis for breeding improved *E. sinensis* species, recovery of the wild *E. sinensis* population and prosperous development of the *E. sinensis* artificial breeding industry.

## 1. Introduction

*Eriocheir sinensis* (Phylum, Arthropod) is a valuable catadromous species found in the Yangtze River of China, the third-largest river in the world. It is distributed worldwide, including in America and Europe, as a result of its strong osmotic adjustment and movement capabilities [1]. The wild *E. sinensis* population has suffered from serious devastation caused by environmental pollution, overconstruction of water conservancy projects and overfishing, among other examples. The Ministry of Agriculture and Rural Affairs of the People’s Republic of China has prohibited commercial fishing of wild *E. sinensis*. The restoration of wild *E. sinensis* resources is being vigorously carried out [2]. In addition, *E. sinensis* is a delicacy and full of nutrients. As an important economic aquatic species, the *E. sinensis* industry has become the pillar of the aquaculture industry [3,4]. Revealing the regulatory mechanisms underlying *E. sinensis* development will lay a theoretical foundation for breeding and releasing juvenile improved *E. sinensis*, thereby promoting the restoration of wild *E. sinensis* resources and the sustainable development of the *E. sinensis* breeding industry.

As an important member of the phylum Arthropoda, *E. sinensis* grows by molting over its whole lifespan. The molting cycle can be divided into four stages according to the morphological characteristics of the setae: post-molt (AB), inter-molt (C), pre-molt (D) and molt (E). During the post-molt stage, water is quickly absorbed, and the exoskeleton gradually hardens. At the inter-molt stage, the exoskeleton continues to harden and mineralize, and the muscle gradually enlarges. At the pre-molt stage, the old skeleton decomposes and is absorbed, and a new skeleton gradually forms [5].

The basic regulatory mechanism of the *E. sinensis* molting process has been revealed; that is, in general, the initiation and termination of molting are coordinated by ecdysone secreted by the Y organ and the molting inhibition hormone (MIH) secreted by the X-organ/sinus gland complex in the eyestalk [6]. In recent years, research on the regulation of *E. sinensis* molting has been extended, mainly regarding the influence and regulatory mechanisms of feed nutrients (gamma-aminobutyric acid, L-tryptophan), culture patterns (stocking density, salinity) and key functional genes (MMP-14 and V-ATPase subunit B) in molting, growth and development [7,8,9,10,11,12]. The results indicated that gamma-aminobutyric acid supplementation can increase food intake. L-tryptophan supplementation plays a positive regulatory role in molting. The optimal stocking density for juvenile and adult *E. sinensis* was 42–85 crabs per m^2^. Low salinity can influence the survival of *E. sinensis*, and high salinity can restrain its embryo development. MMP-14 functions during *E. sinensis* immune response. V-ATPase subunit B plays a regulatory role in osmoregulation and is essential for *E. sinensis* molting [7,8,9,10,11,12]. The central nervous system is an important organ in the regulation of molting, and the neuropeptides synthesized and secreted by the central nervous system of crabs play an important regulatory role in many physiological processes, including ovary maturation and molting growth [13,14]. Nevertheless, there are no reports on the regulatory mechanisms of the nervous system in the *E. sinensis* molting cycle.

As mentioned, research on the mechanisms regulating *E. sinensis* molting is still at the initial stage. High-throughput sequencing is an efficient and essential technique for preliminarily revealing key regulatory pathways and genes involved in this process. Therefore, in this study, a comparative transcriptome analysis of *E. sinensis* thoracic ganglia at post-molt and inter-molt stages was carried out for the first time to reveal key regulatory pathways and functional genes at the post-molt stage. The results form a theoretical foundation for research on the regulatory network of the *E. sinensis* nervous system during the post-molt developmental period, and such details can be used toward breeding improved *E. sinensis* species, the recovery of the wild *E. sinensis* population and the prosperity of the *E. sinensis* artificial breeding industry.

## 2. Materials and Methods

### 2.1. Experimental Crabs and Sample Collection

One-year-old juvenile *E. sinensis* (average body weight was 12.5 ± 0.79 g) crabs were supplied by Jiangs’ Noah’s Ark Agricultural Science and Technology Co., Ltd. (Changzhou, China). Individuals of a similar size and at the same developmental stage were selected and cultured in three aquariums. Ten female *E. sinensis* crabs and the same number of male juveniles were cultured in the same aquarium. The aquariums were continuously aerated, and the water quality was monitored every day, including water temperature, pH, the concentration of dissolved oxygen, NH_3_-N and NO_2_. *E. sinensis* were fed with compound feed twice each day (at 14:00 and 17:00). The molting stage was determined according to the report from Kang et al. [5]. Cameras were installed in each aquarium to monitor molting, and the molting process was observed 24 h each day after ingestion of *E. sinensis* was reduced. The thoracic ganglia were collected within half an hour after molting; one male sample and one female sample at the post-molt stage were collected from each tank. Likewise, we also collected the same number of thoracic ganglia of *E. sinensis* at the inter-molt phase. Body size parameters of sampled *E. sinensis* were measured before sampling.

### 2.2. Total RNA Extraction and ILLUMINA Sequencing

According to the manufacturer’s instructions, total RNA was extracted with RNAiso reagent (TaKaRa, Kusatsu, Japan). Then, equal amounts of total RNA from the thoracic ganglia of one female and one male crab at the same developmental stage in each tank were pooled as one sample. Three samples for the post-molt stage (MP) and three samples for the inter-molt stage (MI) were finally obtained. The RNA samples were checked for quality, and the detailed operation for quantification of extracted total RNA, construction of the cDNA library and high-throughput sequencing was performed according to the methods reported in our previous study [15]. The generated raw data were submitted to NCBI (NCBI, Bethesda, MD, USA) with accession number PRJNA822878.

### 2.3. Data Filtering and Assembly

The raw data were filtered using NGS QC TOOLKIT V2.3.3 software (Roche, NY, USA) and analyzed, and sequences representing low-quality reads, contaminated reads, primers and adapters were removed [16]. The filtered clean data were assembled using Trinity software (v2.2.0, Singapore) [17].

### 2.4. Transcriptome Annotation

The unigenes were aligned in accordance with the following databases: non-redundant protein (Nr), non-redundant nucleotide (Nt), Swiss-Prot (http://www.uniprot.org/downloads) (accessed on 1 May 2002), clusters of orthologous groups for eukaryotic complete genomes (KOG, ftp://ftp.ncbi.nih.gov/pub/COG/KOG/kyva (accessed on 6 June 2002)) and the Kyoto Encyclopedia of Genes and Genomes (KEGG, http://www.genotup/kegg/pathway.html (accessed on 9 March 1995)) [18,19]. Gene ontology (GO) homology annotation was carried out using Blast2GO software (Valencia, Spain) [20].

### 2.5. Differential Expression Analysis

Differential expression analysis was carried out using the DESeq R package (1.18.0) [21]. Fold change was calculated as the ratio of the expression level of genes in the MI sample and MP sample. In addition, |log_2_ fold change| > 1 and *padj* < 0.05 (adjusted *p* value) were set as the cutoff thresholds for differentially expressed genes (DEGs). The detailed method for the differential expression analysis was performed according to our previous study [15]. GO and KEGG enrichment analyses were carried out on DEGs (*padj* < 0.05). Finally, we obtained the top 30 GO terms and top 30 KEGG pathways. The methods are detailed in our previous study [15].

### 2.6. Quantitative Real-Time PCR (qPCR) Validation

The accuracy of high-throughput data was validated using qPCR. Ten DEGs were randomly selected from transcriptome data, and a qPCR experiment was performed on an ABI 7500 real-time PCR system (ABI, Waltham, MA, USA). The primers were designed with Primer Premier 6 software. Beta-actin was used as the internal reference, and the amplifications were performed according to the following program: 95 °C for 30 s and 40 cycles of 95 °C for 5 s, 60 °C for 35 s and 72 °C for 52 s. Sample detection was triplicated, and the gene expression levels were calculated using the 2^−ΔΔCT^ method [22]. Statistical significance (*p* *<* 0.05) was calculated using one-way ANOVA and Duncan’s multiple range tests (SPSS 21.0). The minimum significance level was set to 0.05.

## 3. Results

### 3.1. Sequencing and Assembly of Thoracic Ganglia Transcriptome of E. sinensis

The body size parameters for *E. sinensis* collected at post-molt and inter-molt stages are shown in Table 1. As shown in Table 2, a total of 290,941,648 clean data were generated. Phred quality score was used as an index for the base-calling accuracy and calculated using FastQC software (Babraham, UK) v0.10.1. In this study, a Q30 value larger than 93% indicated that the base-calling accuracy for each replicate had reached 99.9% and met the requirement for further analysis. After assembly, we obtained 40,121 unigenes. Among these, 22,198 unigenes were longer than 500 bp, the max length was longer than 13,628 bp, the average length was 921.66 b, and N50 was 1209 bp.

### 3.2. Top 30 GO Enrichment Analysis on DEGs at Post-Molt and Inter-Molt Stages

**Figure 1:**
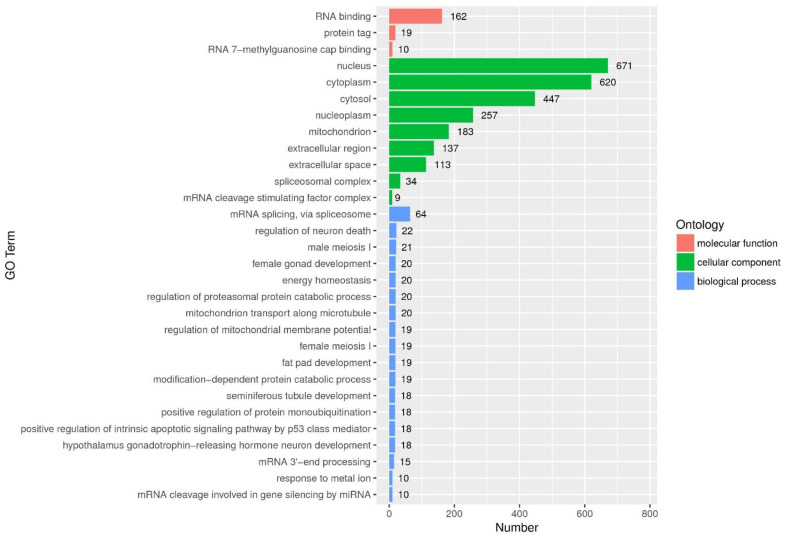
Top 30 GO terms: The horizontal axis indicates gene numbers in each term. The vertical axis indicates top 30 GO terms.

GO defines three levels of ontologies, including molecular function (MC), biological process (BP) and cellular component (CC). As shown in Figure 1, DEGs were mostly enriched in numerous terms in the subcategory of biological process, including development regulation (‘regulation of neuron death’, ‘fat pad development’, ‘seminiferous tubule development’, ‘positive regulation of intrinsic apoptotic signaling pathway by p53 class mediator’, ‘hypothalamus gonadotrophin-releasing hormone neuron development’), energy homeostasis and anti-stress response (‘energy homeostasis’, ‘mitochondrion transport along microtubule’, ‘regulation of mitochondrial membrane potential’) and the regulation of proteometabolism (‘regulation of proteasomal protein catabolic process’, ‘modification-dependent protein catabolic process’, ‘positive regulation of protein monoubiquitination’).

### 3.3. Top 30 KEGG Enrichment Analysis

**Figure 2:**
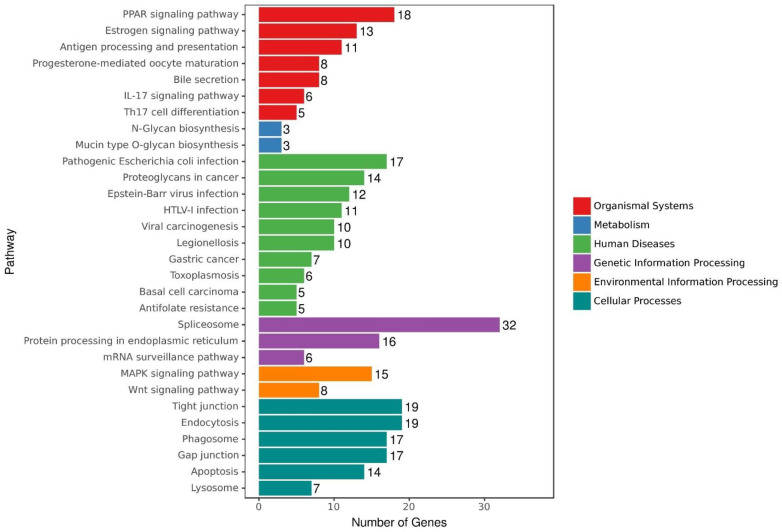
Top 30 KEGG pathways.

As shown in Figure 2, the functions of the identified DEGs were mainly associated with six categories: organismal systems, metabolism, human diseases, genetic information processing, environmental information processing and cellular processes. The top 30 KEGG pathways were mainly relevant to three subcategories, namely the regulation of immune response, energy metabolism (mainly related to glycolysis and lipolysis) and neuronal signal transduction. The major pathways relevant to immune response were ‘antigen processing and presentation’, ‘IL-17 signaling pathway’, ‘Th17 cell differentiation’ and ‘lysosome’. Energy metabolism pathways mainly involved the ‘PPAR signaling pathway’, ‘N-glycan biosynthesis’ and ‘mucin-type O-glycan biosynthesis’. The main pathways related to signal transduction mainly involved the ‘tight junction’, ‘gap junction’, ‘MAPK signaling pathway’, ‘Wnt signaling pathway’ and ‘endocytosis’.

### 3.4. Analysis of Functional DEGs

**Figure 3:**
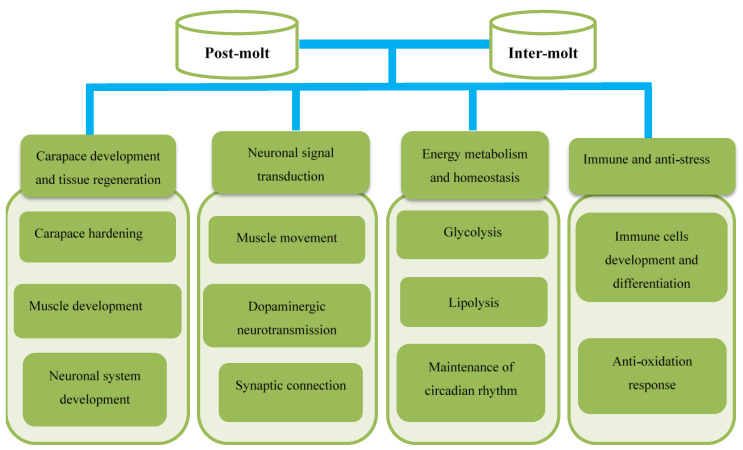
Regulatory network in the thoracic ganglia of *E. sinensis* identified between the post-molt and inter-molt stages. The key regulatory pathways and genes were classified into four categories.

The top 30 GO terms and top 30 KEGG pathways were comprehensively analyzed, and the key DEGs can be classified into four categories: carapace development and tissue regeneration, neuronal signal transduction, energy metabolism and homeostasis maintenance and immune and anti-stress response. The key functional DEGs are shown in Table 3, and all DEGs in this study are shown in [23]. The regulatory network of *E. sinensis* thoracic ganglia is shown in Figure 3.

### 3.5. Validation of Transcriptome Data by qPCR

**Figure 4:**
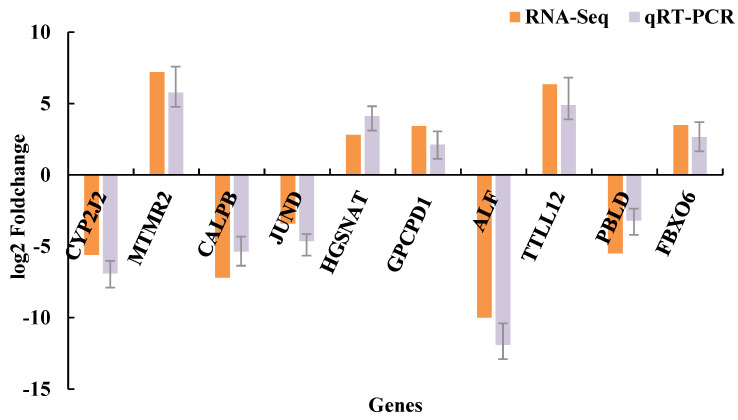
Validation of DEGs by qRT-PCR: The horizontal axis shows gene names. The vertical axis shows relative expression.

Primers for the 10 detected DEGs are shown in [23]. As shown in Figure 4, relative expression levels of the detected DEGs as measured by qPCR were nearly consistent with those determined by high-throughput sequencing. These results indicated that the transcriptome data in this study are reliable.

## 4. Discussion

As shown in Table 3, with the comprehensive analysis of the top 30 GO and top 30 KEGG, differentially expressed KEGG and DEGs can be divided into four categories: carapace development and tissue regeneration, neuronal signal transduction, energy metabolism and homeostasis regulation and immune and anti-stress response.

### 4.1. Carapace Development and Tissue Regeneration at Post-Molt Stage

In this study, some genes relevant to carapace development (bursicon-alpha subunit, *DDC*, *CHS*, *CS*, *BMP* and *BMPR2*), skeletal muscle development (*ACTA1* and *ACTR2*) and neuronal system development (*RERE*, *EPHA2*, *ISWI* and *YAP1*) were significantly differentially expressed. The newly formed soft carapace was gradually hardened under the regulation of various neuropeptides and factors after molting. In this study, some DEGs related to carapace formation were significantly upregulated compared with the pre-molt stage, such as bursicon-alpha subunit, *DDC* and *CHS*. Bursicon is a neuropeptide that has been shown to play a core regulatory role in carapace sclerotization during the post-molt period in the blue crab (*Callinectes sapidus*) and shore crab (*Carcinus maenas*) [24,25]. *DDC* catalyzes the conversion of dopa into dopamine and then initiates the subsequent process of exoskeleton sclerotization and mineralization [26]. *CS* catalyzes chitin biosynthesis and plays a regulatory role in the development of the cuticular layer [27]. In this study, *CS* was significantly upregulated to promote the formation of the new carapace. In addition, some regulatory genes relevant to bone development were also upregulated, such as *BMP* and *BMPR2* after molting. The research on the regulatory pattern of the Y organs of the blackback land crab (*Gecarcinus lateralis*) during the molt cycle also indicated that some genes related to bone formation, such as BMP7 and BMP receptor 1B, were upregulated during the post-molt stage [6]. BMPs, comprising a superfamily of growth factors, are closely associated with animal growth and development, and most members of the BMP family have been shown to play an important regulatory role in biocalcification, tissue reconstruction and regeneration [28]. BMP, an important member of the TGF-beta superfamily, can activate *BMPR2* and participate in BMP signaling [29].

In addition, some genes relevant to skeletal muscle development were also upregulated at the post-molt stage, such as *ACTA1* and *ACTR2*. Alpha-actin, a major contractile constituent of skeleton muscle, plays a key regulatory role in the mediation of actin networks [30]. Research on the molting regulation of *Litopenaeus vannamei* indicates that some actin-related regulatory genes are upregulated after molting, such as skeletal muscle actin 6 [31]. The post-molt period is an important stage for muscle regeneration and morphological remodeling.

In this study, some novel genes functioning in the regulation of the neuronal system and connective tissue development were significantly upregulated, such as *RERE*, *EPHA2*, *ISWI* and *YAP1*. RERE acts as a transcriptional repressor for cell survival and development [32]. Its downregulation as observed in this study is expected to promote post-molt development of *E. sinensis*. *EPHA2* functions in the regulation of brain development and angiogenesis [33]. *ISWI* plays a pivotal regulatory part in larval blood cell development and metamorphosis [34]. *YAP1*, a transcriptional regulator, plays a critical role in the regulation of tissue tension and shape [35].

### 4.2. Neuronal Signal Transduction after Molting

In this study, some key regulatory genes relevant to signal transduction were upregulated, such as neuroparsin and tachykinin. Neuroparsin can induce the elevation of trehalose and hemolymph lipids. It was first identified as a regulatory factor functioning in ovary maturation [36,37]. In this study, neuroparsin-2 was significantly elevated after molting. A study on the regulatory function of neuropeptides in green shore crabs during the molt cycle showed that neuroparsin-1 is involved in regulation during the molt cycle [38]. The specific biological function of neuroparsin at the post-molt developmental stage of *E. sinensis* remains to be elucidated through further study. Tachykinins are excitatory neuropeptides that can initiate the contraction of multiple smooth muscles, and they mediate the downstream neuronal signaling pathway together with tachykinin receptors [39,40]. In this study, tachykinin receptors, such as *TKR99D* and *TKR86C*, were upregulated after molting; similarly, tachykinin upregulation was observed in a transcriptomic analysis study of neuropeptidome in lobster *Homarus americanus* eyestalk ganglia [41]. In addition, other regulatory genes related to neuronal signal transduction were also upregulated, including *PPP1R9B*, *PlEXB* and *RICH*. *PPP1R9B* plays a pivotal regulatory role in dopaminergic neurotransmission [42]. *PlEXB* participates in axon guidance of the neuronal system [43]. *RICH* is an indispensable regulator for the formation of synaptic connections [44].

### 4.3. Regulation of Energy Metabolism and Homeostasis

In this study, regulatory genes relevant to glycan synthesis, such as *UXS1*, were downregulated after molting. Some genes related to lipid catabolism genes (*ENGASE*, *MTTP* and *PNLIPRP2*) and homeostasis regulation (*HNF4A*, *RGN* and *TIM*) were upregulated. A study on the energy metabolism of *E. sinensis* hepatopancreas and its association with molting indicated that genes relevant to glycolysis and lipolysis are upregulated during the post-molt stage [45]. *UXS1*, a catalyst for decarboxylation of UDP-glucuronic acid, is indispensable in tetrasaccharide biosynthesis [46]. In our study, *UXS1* was found to be downregulated, which is beneficial for glycolysis and energy supply. *ENGASE* plays a regulatory role in the release of N-glycans from glycoprotein [47] and was upregulated for energy supply in our study. *MTTP* can regulate the biosynthesis of cholesteryl ester [48]. *PNLIPRP2*, as a lipase, participates in the hydrolyzation of triglycerides [49]. *HNF4A*, as a transcription factor, functions in the maintenance of the circadian rhythm of liver genes [50]. *RGN* can modulate Ca^2+^-dependent enzyme activities and is essential for the maintenance of calcium homeostasis [51]. *TIM* can determine the formation of circadian rhythm together with period circadian protein [52].

The post-molt stage is a period for carapace development and tissue regeneration, during which a sufficient energy supply is required, and glycolysis and lipolysis are vigorous. Genes related to homeostasis regulation were upregulated for the maintenance of homeostasis of signal transduction and energy metabolism during the post-molt phase.

### 4.4. Regulation of Immune and Anti-Stress Response

In this study, some immune-relevant regulatory genes involved in immune cell response and antigen recognition were upregulated after molting, such as *Frizzle1/7*, *SMPDL3B*, *FCN1* and *ITGA4*. Furthermore, some modulatory genes related to anti-stress response were also significantly expressed, including *GCLC* and *KEAP1*. The Wnt signaling pathway plays a critical regulatory role in intercellular development and differentiation of macrophages, T cells, B cells, etc. Frizzled is the receptor of Wnt protein, and it plays a positive regulatory role in immune response [53]. In this study, *Frizzled 1* and *Frizzled 7* were upregulated. Similarly, the transcriptome analysis of the Y organs of blackback land crabs showed that Frizzled plays an important role in the immune response during the molting cycle [54]. *SMPDL3B*, which is located on the surface of macrophages and dendritic cells, plays a vital role in the lipid composition of macrophages and a negative role in immunity [55]. Its downregulation, as observed in this study, is beneficial to immunity enhancement. *FCN1* plays a pivotal regulatory role in the activation of the lectin pathway of the complement system [56]. *ITGA4* can initiate leukocyte aggregation for an immune response [57]. Glutathione, as an important intracellular regulator, plays an important role in the maintenance of the immune system and functions in antioxidant and detoxification processes [58]. *GCLC*, as a key component of glutamate–cysteine ligase, participates in the rate-limiting step of glutathione biosynthesis [59]. *KEAP1*, as a pivotal transcription factor, plays an important regulatory role in antioxidation responses [60].

Many genes relevant to the regulation of the immune response were found to be upregulated. The reason for this is that *E. sinensis* is vulnerable to the invasion of pathogens during the post-molt period. At this time, the hard exoskeleton is not well-formed, and thus, immunity is enhanced to protect *E. sinensis* from harm. Discerning the details of the specific regulatory mechanisms awaits further study.

### 4.5. Application of This Study

In this study, four types of key functional genes were differentially expressed during the *E. sinensis* post-molt stage: These genes can be screened for breeding improved varieties of *E. sinensis*. In the near future, transgenic technology can be applied in the *E. sinensis* industry, and the characteristic improvement of *E. sinensis* can be performed from four aspects, namely carapace development, tissue regeneration, energy homeostasis maintenance and immune response. The obtained improved *E. sinensis* with a faster growth rate and stronger resistance can provide high-quality juvenile crabs for the breeding industry of *E. sinensis* and the proliferation and release of wild *E. sinensis*, thus promoting the recovery of wild *E. sinensis* resources.

## 5. Conclusions

In this study, a comparative transcriptome study of *E. sinensis* thoracic ganglia at the post-molt and inter-molt stages was carried out for the first time. The results indicate that pathways and regulatory genes related to carapace development, tissue regeneration, glycolysis and lipolysis and immune and anti-stress responses were significantly differentially expressed. At present, research on the regulatory mechanisms of the nervous system in *E. sinensis* during the molting cycle period is scarce. The results of this study lay a theoretical foundation for research on the regulatory networks operating in the nervous system of *E. sinensis* during the post-molt developmental period. Detailed knowledge of the regulatory network involved in *E. sinensis* molting can be used for breeding improved *E. sinensis* species, the recovery of the wild *E. sinensis* population and the lucrative development of the *E. sinensis* artificial breeding industry.

## Figures and Tables

**Table 1:** Parameter for body size of sampled *E. sinensis*.

ID	Weight (g)	Carapace Length (mm)	Carapace Width (mm)
MP1-F	11.7	26.9	27.8
MP1-M	12.1	27.6	28.1
MP2-F	11.2	26.8	27.5
MP2-M	12.4	27.9	28.7
MP3-F	11.1	26.5	27.6
MP3-M	12.2	26.6	28.5
MI1-F	11.6	26.8	27.3
MI1-M	13.2	27.9	28.5
MI2-F	11.5	26.7	27.3
MI2-M	12.5	27.7	28.8
MI3-F	11.4	26.5	27.4
MI3-M	12.9	27.5	28.1

Note: MP1-F~MP3-F: three female *E. sinensis* at post-molt stage in three aquariums; MP1-M~MP3-M: three male *E. sinensis* at post-molt stage in three aquariums; MI1-F~MI3-F: three female *E. sinensis* at inter-molt stage in three aquariums; MI1-M~MI3-M: three male *E. sinensis* at inter-molt stage in three aquariums.

**Table 2:** Summary of sequencing of thoracic ganglia transcriptome of *E. sinensis*.

Sample	Raw Reads	Raw Bases	Clean Reads	Clean Bases	Q20 (%)	GC (%)
MP1	45,960,600	6,894,090,000	45,473,604	6,728,590,578	95.9	50.1
MP2	45,521,480	6,828,222,000	45,188,428	6,707,176,823	96.1	49.8
MP3	45,570,666	6,885,599,900	44,245,592	6,711,431,282	95.2	50.6
MI1	45,859,836	6,893,975,400	44,236,908	6,749,106,113	96.6	49.9
MI2	46,045,208	6,951,781,200	45,992,414	6,861,547,596	96.3	49.8
MI3	45,360,592	6,859,088,800	45,161,228	6,743,440,718	96.1	50.9

Note: MP1–3: three replicates of thoracic ganglia of post-molt *E. sinensis*; MI1–3: three replicates of thoracic ganglia of inter-molt *E. sinensis*; valid bases: valid base ratio; Q20: ratio of bases with Phred quality score larger than 20 in raw bases.

**Table 3:** Key DEGs in thoracic ganglia transcriptome of *E. sinensis*.

Category	Gene Name	Gene Definition	log_2_ Fold Change	*padj*
Carapace development and tissue regeneration	*Bursicon-α*	Bursicon alpha	−3.35	0.02
	*DDC*	Dopa decarboxylase	−5.46	0.05
	*CHS*	Chitin synthase	−2.02	0.01
	*BMP*	Bone morphogenetic protein type II	−1.73	0.02
	*BMPR2*	Bone morphogenetic protein receptor type-2	−5.66	0.04
	*ACTA1*	Alpha actin 1	−1.70	0.03
	*ACTR2*	Actin-related protein 2	−1.89	0.05
	*RERE*	Arginine-glutamic acid dipeptide repeats protein	−3.23	0.02
	*EPHA2*	Ephrin type-A receptor 2	−3.86	0.01
	*ISWI*	Chromatin-remodeling complex ATPase chain Iswi	−2.41	0.04
	*NEFH*	Neurofilament heavy polypeptide	−3.18	0
	*YAP1*	Transcriptional coactivator YAP1	−2.44	0.01
	*ADAMTS3*	A disintegrin and metalloproteinase with thrombospondin motifs 3	−2.04	0.01
Neuronal signal transduction	*OEH*	Neuroparsin 2	−1.60	0.04
	*TKR86C*	Tachykinin-like peptides receptor 86C	−1.90	0.04
	*TKR99D*	Tachykinin-like peptides receptor 99D	−2.42	0.04
	*PPP1R9B*	Neurabin-2	−5.79	0.03
	*PLEXB*	Plexin-B	−1.95	0.02
	*PICK1*	PRKCA-binding protein	−5.48	0.05
	*FLNB*	Filamin-B	−2.12	0.02
	*RICH*	Guanine nucleotide exchange factor subunit Rich	−4.77	0.02
Energy metabolism and homeostasis maintenance	*UXS1*	UDP-glucuronic acid decarboxylase 1	2.64	0.04
	*ENGASE*	Cytosolic endo-beta-N-acetylglucosaminidase	−5.63	0.04
	*NOCT*	Nocturnin	−2.46	0.04
	*MTTP*	Microsomal triglyceride transfer protein large subunit	−4.81	0
	*PNLIPRP2*	Pancreatic lipase-related protein 2	−2.26	0.04
	*HNF4A*	Hepatocyte nuclear factor 4-alpha	−2.19	0.04
	*RGN*	Regucalcin	−2.62	0
Immune and anti-stress response	*FZD1*	Frizzled-1	−3.76	0.04
	*FZD7*	Frizzled-7	−1.91	0.02
	*SMPDL3B*	Acid sphingomyelinase-like phosphodiesterase 3b	−3.32	0.04
	*FCN1*	Ficolin-1	−2.01	0.02
	*ITGA4*	Integrin alpha-4	−2.38	0.05
	*GCLC*	Glutamate–cysteine ligase catalytic subunit	−2.80	0
	*KEAP1*	Kelch-like ECH-associated protein 1	−3.57	0

Note: *padj*: adjusted *p*-value.

## Data Availability

Data can be available in reference [23].

## Abbreviations

*E. sinensis*, *Eriocheir sinensi**s*; MIH, molting inhibition hormone; MP, post-molt stage; MI, inter-molt stage; DEGs, differentially expressed genes; molecular function (MC), biological process (BP) and cellular component (CC).

## References

[1] Spiridonov V.A., Zalota A.K. (2017). Understanding and forecasting dispersal of non-indigenous marine decapods (Crustacea: Decapoda) in East European and North Asian waters. J. Mar. Biol. Assoc. UK.

[2] Wang H.H., Zhuang P., Feng G.P., Gao Y., Zhao F.S. (2016). Study on resources dynamics and conservation of *Eriocheir sinensis* in the middle and lower reaches of the Yangtze River. Acta Agric. Zhejiangensis.

[3] Wang Q., Wu X., Long X., Zhu W., Cheng Y. (2018). Nutritional quality of different grades of adult male chinese mitten crab, *Eriocheir sinensis*. J. Food Sci. Technol..

[4] Song Q.H., Zhao Y.F. (2018). Analysis on Current Situation and Criteria for *Eriocheir sinensis* Culturing Industry. Sci. Fish Farming.

[5] Kang X.J., Tian Z.H., Wu J.L., Mu S.M. (2012). Molt stages and changes in digestive enzyme activity in hepatopancreas during molt cycle of Chinese mitten crab, *Eriocheir sinensis*. J. Fish. Sci. China.

[6] Das S., Vraspir L., Zhou W., Durica D.S., Mykles D.L. (2018). Transcriptomic analysis of differentially expressed genes in the molting gland (Y-organ) of the blackback land crab, Gecarcinus lateralis, during molt-cycle stage transitions. Comp. Biochem. Physiol. Part D Genom. Proteom..

[7] Zhang C., Wang X.D., Su R.Y., He J.Q., Liu S.B., Huang Q.C., Qin C.J., Zhang M.L., Qin J.G., Chen L.Q. (2022). Dietary gamma-aminobutyric acid (GABA) supplementation increases food intake, influences the expression of feeding-related genes and improves digestion and growth of Chinese mitten crab (*Eriocheir sinensis*). Aquaculture.

[8] Zhang C., Zhang J., Huang G., Xu M., Cheng Y., Yang X. (2021). Effects of dietary L-tryptophan supplementation on growth performance, food intake, digestive enzyme activity and serotonin (5-HT) levels in juvenile Chinese mitten crab (*Eriocheir sinensis*). Aquacult. Nutr..

[9] Yuan Q., Qian J., Ren Y., Zhang T.L., Li Z.J., Liu J.S. (2018). Effects of stocking density and water temperature on survival and growth of the juvenile Chinese mitten crab, *Eriocheir sinensis*, reared under laboratory conditions. Aquaculture.

[10] Wang R.F., Huang X.R., Wang H.H., Lu J.X., Shi X.T., Feng G.P., Zhuang P. (2019). Effects of salinity on embryonic and larval development of Chinese mitten crab *Eriocheir sinensis* (Decapoda: Brachyura) and salinity-induced physiological changes. J. Oceanol. Limnol..

[11] Li R., Meng Q.H., Huang J.W., Wang S., Sun J.S. (2020). MMP-14 regulates innate immune responses to *Eriocheir sinensis* via tissue degradation. Fish Shellfish Immunol..

[12] Hou X., Chen X.W., Yang H., Yue W.C., Wang J., Han H., Wang C.H. (2020). V-ATPase subunit B plays essential roles in the molting process of the Chinese mitten crab, *Eriocheir sinensis*. Biol. Open.

[13] Liu X., Ma K., Liu Z., Feng J., Ye B., Qiu G. (2019). Transcriptome analysis of the brain of the Chinese mitten crab, *Eriocheir sinensis*, for neuropeptide abundance profiles during ovarian development. Anim. Reprod. Sci..

[14] Das S., Mykles D.L. (2016). A comparison of resources for the annotation of a de novo assembled transcriptome in the molting gland (Y-Organ) of the blackback land crab, *Gecarcinus lateralis*. Integr. Comp. Biol..

[15] Wang M.Y., Tang Y.K., Yu J.H., Su S.Y., Li J.L., Yu F., Li H.X., Song C.Y., Du F.K., Xu P. (2020). Molecular insights into the sex-differential regulation of signal transduction in the cerebral ganglion and metabolism in the hepatopancreas of *Eriocheir sinensis* during reproduction. Genomics.

[16] Patel R.K., Jain M. (2012). NGS QC Toolkit: A toolkit for quality control of next generation sequencing data. PLoS ONE.

[17] Grabherr M.G., Haas B.J., Yassour M., Levin J.Z., Thompson D.A., Amit I., Adiconis X., Fan L., Raychowdhury R., Zeng Q. (2011). Full-length transcriptome assembly from RNA-Seq data without a reference genome. Nat. Biotechnol..

[18] Altschul S.F., Gish W., Miller W., Myers E.W., Lipman D.J. (1990). Basic local alignment search tool. J. Mol. Biol..

[19] Kanehisa M., Araki M., Goto S., Hattori M., Hirakawa M., Itoh M., Katayama T., Kawashima S., Okuda S., Tokimatsu T. (2008). KEGG for linking genomes to life and the environment. Nucleic Acids Res..

[20] Conesa A., Gotz S., Garcia J. (2005). Blast2GO: A universal tool for annotation, visualization and analysis in functional genomics research. Bioinformatics.

[21] Anders S., Huber W. (2012). Differential Expression of RNA-Seq Data at the Gene Level—The DESeq Package.

[22] Livak K., Schmittgen T.D. (2001). Analysis of relative gene expression data using real-time quantitative PCR and the 2^–ΔΔCT^ method. Methods.

[23] Wang M. Comparative transcriptome analysis on nervous system of *E. sinensis* during molting cycle. Mendeley Data. 2022, V1. https://data.mendeley.com/datasets/hnpy5vvhyh/1.

[24] Chung J.S., Katayama H., Dircksen H. (2012). New functions of arthropod bursicon: Inducing deposition and thickening of new cuticle and hemocyte granulation in the blue crab, *Callinectes sapidus*. PLoS ONE.

[25] Webster S.G., Wilcockson D.C., Sharp J.H. (2013). Bursicon and neuropeptide cascades during the ecdysis program of the shore crab, *Carcinus maenas*. Gen. Comp. Endocrinol..

[26] Christie A.E. (2019). Identification of putative amine biosynthetic enzymes in the nervous system of the crab, *Cancer borealis*. Invertebr. Neurosci..

[27] Schmid S., Song Y., Tollefsen K.E. (2021). AOPReport: Inhibition of chitin synthase1leading to increased mortality in arthropods. Environ. Toxicol. Chem..

[28] Fan S.G., Zhou D.Z., Liu B.S., Deng Z.H., Guo Y.H., Yu D.H. (2018). Molecular cloning and expression analysis of BMP7b from *Pinctada fucata*. South China Fish. Sci..

[29] Khodr V., Machillot P., Migliorini E., Reiser J.B., Picart C. (2021). High-throughput measurements of bone morphogenetic protein/bone morphogenetic protein receptor interactions using biolayer interferometry. Biointerphases.

[30] Uddowla M.H., Salma U., Kim H.W. (2013). Molecular characterization of four actin cDNAs and effects of 20-hydroxyecdysone on their expression in swimming crab, *Portunus trituberculatus* (Miers, 1876). Anim. Cells Syst..

[31] Gao Y., Zhang X.J., Wei J.K., Sun X.Q., Yuan J.B., Li F.H., Xiang J.H. (2015). Whole transcriptome analysis provides insights into molecular mechanisms for molting in *Litopenaeus vannamei*. PLoS ONE.

[32] Kim B.J., Scott D.A. (2014). Mouse model reveals the role of RERE in cerebellar foliation and the migration and maturation of purkinje cells. PLoS ONE.

[33] Zhou Y., Bennett T.M., Ruzycki P.A., Shiels A. (2021). Mutation of the EPHA2 tyrosine-kinase domain dysregulates cell pattern formation and cytoskeletal gene expression in the Lens. Cells.

[34] Jakada B.H., Aslam M., Fakher B., Greaves J.G., Li Z., Li W., Lai L., Ayoade O.A., Cheng Y., Cao S. (2019). Identification of SWI2/SNF2-Related 1 Chromatin Remodeling Complex (SWR1-C) Subunits in Pineapple and the Role of Pineapple SWR1 COMPLEX 6 (AcSWC6) in Biotic and Abiotic Stress Response. Biomolecules.

[35] Stein C., Bardet A.F., Roma G., Bergling S., Clay I., Ruchti A., Agarinis C., Schmelzle T., Bouwmeester T., Schubeler D. (2015). YAP1 Exerts Its Transcriptional Control via tead-mediated activation of enhancers. PLoS Genet..

[36] Ye H., Wilder M.N., Dircksen H., Chung J.S. (2021). Editorial: Recent advances in crustacean endocrinology. Front. Endocrinol..

[37] Yang S.P., He J.G., Sun C.B., Chan S.F. (2014). Characterization of the shrimp neuroparsin (MeNPLP): RNAi silencing resulted in inhibition of vitellogenesis. Febs Open Bio.

[38] Oliphant A., Alexander J.L., Swain M.T., Webster S.G., Wilcockson D.C. (2018). Transcriptomic analysis of crustacean neuropeptide signaling during the moult cycle in the green shore crab, *Carcinus maenas*. BMC Genom..

[39] DeMaegd M.L., Stein W. (2021). Neuropeptide modulation increases dendritic electrical spread to restore neuronal activity disrupted by temperature. J. Neurosci..

[40] Rainey A.N., Fukui S.M., Mark K., King H.M., Blitz D.M. (2021). Intrinsic sources of tachykinin-related peptide in the thoracic ganglion mass of the crab, *Cancer borealis*. Gen. Comp. Endocrinol..

[41] Christie A.E., Roncalli V., Cieslak M.C., Pascual M.G., Yu A., Lameyer T.J., Stanhope M.E., Dickinson P.S. (2017). Prediction of a neuropeptidome for the eyestalk ganglia of the lobster Homarus americanus using a tissue-specific de novo assembled transcriptome. Gen. Comp. Endocrinol..

[42] Foley K., McKee C., Nairn A.C., Xia H. (2021). Regulation of synaptic transmission and plasticity by protein phosphatase 1. J. Neurosci..

[43] Van Battum E., Heitz-Marchaland C., Zagar Y., Fouquet S., Kuner R., Chedotal A. (2021). Plexin-B2 controls the timing of differentiation and the motility of cerebellar granule neurons. eLife.

[44] Wei J., Jia M., Zhang C., Wang M., Gao F., Xu H., Gong W. (2010). Crystal structure of the C-terminal domain of the epsilon subunit of human translation initiation factor eIF2B. Protein Cell.

[45] Huang S., Wang J., Yue W., Chen J., Gaughan S., Lu W., Lu G., Wang C. (2015). Transcriptomic variation of hepatopancreas reveals the energy metabolism and biological processes associated with molting in Chinese mitten crab, *Eriocheir sinensis*. Scie. Rep..

[46] Eixelsberger T., Sykora S., Egger S., Brunsteiner M., Kavanagh K.L., Oppermann U., Nidetzky B. (2012). Structure and Mechanism of Human UDP-xylose Synthase evidence for a promoting role of sugar ring distortion in a three-step catalytic conversion of UDP-glucuronic acid. J. Biol. Chem..

[47] Maeda M., Okamoto N., Araki N., Kimura Y. (2021). Purification, characterization, and gene expression of rice endo-beta-N-acetylglucosaminidase, Endo-Os. Front. Plant Sci..

[48] Biterova E.I., Isupov M.N., Keegan R.M., Lebedev A.A., Sohail A.A., Liaqat I., Alanen H.I., Ruddock L.W. (2019). The crystal structure of human microsomal triglyceride transfer protein. Proc. Natl. Acad. Sci. USA.

[49] Sahaka M., Amara S., Wattanakul J., Gedi M.A., Aldai N., Parsiegla G., Lecomte J., Christeller J.T., Gray D., Gontero B. (2020). The digestion of galactolipids and its ubiquitous function in Nature for the uptake of the essential alpha-linolenic acid. Food Funct..

[50] Qu M., Duffy T., Hirota T., Kay S.A. (2018). Nuclear receptor HNF4A transrepresses clock: BMAL1 and modulates tissue-specific circadian networks. Proc. Natl. Acad. Sci. USA.

[51] Singh T., Banerjee P., Uditi, Kumari S., Chopra A., Singh N., Qamar I. (2022). Expression of Regucalcin, a calcium-binding protein is regulated by hypoxia-inducible factor-1alpha. Life Sci..

[52] Deppisch P., Prutscher J.M., Pegoraro M., Tauber E., Wegener C., Helfrich-Foerster C. (2022). Adaptation of *Drosophila melanogaster* to long photoperiods of high-latitude summers is facilitated by the ls-timeless allele. J. Biol. Rhythm..

[53] Bats M., Peghaire C., Delobel V., Dufourcq P., Couffinhal T., Duplaa C. (2022). Wnt/frizzled signaling in endothelium: A major player in blood-retinal- and blood-brain-barrier integrity. Cold Spring Harb. Perspect. Med..

[54] Tran N.M., Mykles D.L., Elizur A., Ventura T. (2019). Characterization of G-protein coupled receptors from the blackback land crab Gecarcinus lateralis Y organ transcriptome over the molt cycle. BMC Genom..

[55] Heinz L.X., Baumann C.L., Koberlin M.S., Snijder B., Gawish R., Shui G.H., Sharif O., Aspalter I.M., Muller A.C., Kandasamy R.K. (2015). The lipid-modifying enzyme SMPDL3B negatively regulates innate immunity. Cell Rep..

[56] Genster N., Ma Y.J., Munthe-Fog L., Garred P. (2014). The pattern recognition molecule ficolin-1 exhibits differential binding to lymphocyte subsets, providing a novel link between innate and adaptive immunity. Mol. Immunol..

[57] Fu Y.W., Chen W.F., He M.H., Tang L., Guo S.Q., Zhang Q.Z. (2022). An integrin alpha 4 (ChInt alpha 4) from oyster Crassostrea hongkongensis mediates the hemocytes phagocytosis towards *Vibrio alginolyticus*. Fish Shellfish Immunol..

[58] Liu K., Liu J., Ren T., Xu Y., Lu M., Fang H., Zhang Y., Liao Y., Zhu P. (2021). Cloning and analysis of three glutathione S-transferases in Eriocheir hepuensis and their expression in response to azadirachtin stress. Aquacult. Rep..

[59] Franklin C.C., Backos D.S., Mohar I., White C.C., Forman H.J., Kavanagh T.J. (2009). Structure, function, and post-translational regulation of the catalytic and modifier subunits of glutamate cysteine ligase. Mol. Aspects Med..

[60] Deng H. (2014). Multiple roles of Nrf2-Keap1 signaling. Fly.

